# Special Issue “Immune-Inflammatory and Oxidative Stress Signaling Pathways Involved in Human Disorders”

**DOI:** 10.3390/ijms27031467

**Published:** 2026-02-02

**Authors:** Emanuela Esposito, Alessio Ardizzone

**Affiliations:** 1 Department of Chemical, Biological, Pharmaceutical and Environmental Sciences, University of Messina, Viale Ferdinando Stagno d’Alcontres 31, 98166 Messina, Italy; eesposito@unime.it; 2 Departmental Faculty of Medicine, UniCamillus-Saint Camillus International University of Health Sciences, Via di Sant’Alessandro 8, 00131 Rome, Italy

Inflammation represents a fundamental and evolutionarily conserved biological response to harmful stimuli, including pathogens, environmental particles, and tissue injury [1]. Under physiological conditions, inflammatory signaling is tightly regulated and instrumental in restoring tissue homeostasis [2].

However, when dysregulated or persistent, inflammation shifts from being a protective mechanism to a driver of pathological processes, contributing to tissue damage, immune dysfunction, and disease progression [3].

Mounting evidence indicates that chronic immune-inflammatory activation rarely acts in isolation [4,5]. Instead, it is intimately intertwined with oxidative stress and redox imbalance, creating a self-perpetuating network of molecular events that disrupt cellular signaling, mitochondrial function, and genomic stability [6]. This maladaptive crosstalk between inflammation, impaired immune responses, and oxidative stress has emerged as a unifying pathogenic framework underlying a wide spectrum of human disorders, ranging from neurodegenerative and neurotraumatic conditions to cardiovascular diseases, cancer, and immune-mediated pathologies [7,8,9,10].

In recent years, advances in molecular biology and translational research have significantly expanded our understanding of the signaling pathways governing immune-inflammatory and oxidative stress responses. Nonetheless, the complexity and context-dependent nature of these pathways continue to pose major challenges for therapeutic intervention. In particular, the identification of reliable molecular biomarkers and actionable targets capable of modulating these interconnected processes remains an unmet need in both preclinical and clinical settings.

Therefore, this Special Issue, “Immune-Inflammatory and Oxidative Stress Signaling Pathways Involved in Human Disorders”, was conceived to provide an integrated and up-to-date overview of the molecular mechanisms linking inflammation, immune dysregulation, and oxidative stress across different disease contexts.

The original research contributions included in this Special Issue provide compelling experimental evidence supporting the central role of immune-inflammatory signaling and oxidative stress as converging mechanisms across distinct pathological conditions. Despite focusing on different disease contexts, both studies highlight how dysregulated cytokine networks, redox imbalance, and immune cell activation critically shape disease progression and severity.

In the oncological setting, the cross-sectional study on Helicobacter pylori-associated gastric adenocarcinoma offers a comprehensive evaluation of systemic inflammatory and oxidative stress profiles across different disease stages. By analyzing a large cohort of patients, Smirnova et al. demonstrate that tumor progression is accompanied by stage-specific alterations in circulating pro- and anti-inflammatory cytokines, lipid peroxidation markers, and antioxidant defense systems [11]. These findings reinforce the concept that chronic infection-driven inflammation and oxidative stress do not merely coexist with gastric cancer but dynamically evolve alongside disease advancement, potentially influencing tumor biology and patient outcomes. Importantly, this work supports the rationale for therapeutic strategies aimed at simultaneously modulating cytokine signaling and oxidative stress pathways in gastric malignancies.

Complementing these clinical observations, the experimental study focusing on Parkinson’s disease explores the pathogenic contribution of the chemokine receptor CCR1 in neuroinflammatory processes. Using a well-established mouse model of nigrostriatal degeneration, the authors demonstrated that pharmacological antagonism of CCR1 markedly attenuates neurodegeneration and motor dysfunction [12]. This neuroprotective effect was associated with a robust reduction in glial activation, NF-κB signaling, pro-inflammatory enzyme expression, and peripheral immune cell infiltration, alongside a normalization of inflammatory cytokine levels. Notably, the increase in circulating CCR1 observed following neurotoxic insult further supports its potential role as a disease-associated biomarker, positioning CCR1 as a promising therapeutic target for immunomodulatory approaches in neurodegenerative disorders.

Taken together, these original studies exemplify how immune-inflammatory and oxidative stress pathways act as shared molecular denominators across seemingly disparate diseases, underscoring the translational relevance of targeting these interconnected mechanisms in both peripheral and central nervous system pathologies.

The two review articles included in this Special Issue further expand the conceptual framework linking immune-inflammatory signaling to disease pathogenesis, offering complementary perspectives that span from systemic cardiovascular disorders to localized bone pathology driven by molecular dysregulation.

The review on atherosclerosis provides a comprehensive and clinically oriented overview of inflammation as a central driver of disease initiation and progression. By tracing the evolution of atherosclerotic lesions from subclinical stages to overt cardiovascular events, Madaudo and colleagues clearly illustrate how chronic inflammatory signaling orchestrates endothelial dysfunction, plaque formation, and instability [13]. Particular attention is devoted to carotid atherosclerosis, emphasizing its epidemiological relevance and its association with cognitive decline, thereby reinforcing the concept of atherosclerosis as a multisystem disease rather than a purely vascular condition. Importantly, the review bridges pathogenic mechanisms with clinical practice, discussing preventive strategies, lifestyle interventions, and therapeutic decision-making, highlighting inflammation as a modifiable target in cardiovascular risk management.

From a more molecular standpoint, the second review focuses on the emerging role of non-coding RNAs in the pathogenesis of osteonecrosis of the jaw. The authors provide a detailed analysis of how inflammatory signaling pathways involved in bone remodeling intersect with epigenetic and post-transcriptional regulatory mechanisms [14]. MicroRNAs, long non-coding RNAs, and circular RNAs are discussed as key modulators of osteoclast and osteoblast function, apoptosis, and tissue degeneration. Beyond their mechanistic relevance, these non-coding RNA species are proposed as potential biomarkers and therapeutic targets, opening new avenues for early diagnosis and personalized interventions in patients at risk of this debilitating condition.

Together, these reviews underscore how immune-inflammatory pathways operate across different biological scales, from systemic chronic diseases to localized tissue-specific disorders, and highlight the importance of integrating molecular insights with clinical strategies to effectively address inflammation-driven pathologies.

This Special Issue was further enriched by a study protocol addressing the emerging role of the gut microbiota in shaping immune-inflammatory and oxidative stress responses in chronic intestinal disorders and colorectal cancer. Focusing on Fusobacterium nucleatum, an increasingly recognized pathogenic contributor to both inflammatory bowel disease and colorectal carcinogenesis, the proposed clinical trial adopts an integrative and multidisciplinary approach.

The protocol is designed to systematically investigate the association between Fusobacterium nucleatum burden and key molecular pathways involved in inflammation, oxidative stress, and tumor progression [15]. By combining tissue-based microbial quantification with the assessment of inflammasome activation, cytokine release, lipid peroxidation markers, and autophagic processes, the study aims to delineate how microbial-driven immune activation contributes to disease severity and therapeutic resistance. The inclusion of radiomic analyses represents an additional innovative aspect, with the potential to correlate imaging phenotypes with molecular and microbial signatures, thereby enhancing disease stratification and non-invasive monitoring. Although primarily methodological in nature, the protocol is supported by preliminary in vitro evidence demonstrating that exposure to Fusobacterium nucleatum adhesins promotes a pro-inflammatory cellular phenotype.

Overall, the contributions gathered in this Special Issue collectively reinforce the concept that immune-inflammatory signaling and oxidative stress represent interconnected and dynamic drivers of human pathology rather than isolated biological phenomena. Across oncological, neurodegenerative, cardiovascular, skeletal, and gastrointestinal diseases, the studies presented herein converge on shared molecular pathways that govern disease initiation, progression, and therapeutic responsiveness.

By integrating clinical observations with experimental models and molecular analyses, this Special Issue underscores the translational value of targeting inflammation and oxidative stress as common denominators across diverse disease contexts. Furthermore, the identification of novel biomarkers and regulatory mechanisms, including chemokine receptors, redox markers, and non-coding RNAs, opens new avenues for precision medicine and personalized therapeutic strategies.

Looking forward, the challenge will be to translate these mechanistic insights into clinically actionable interventions capable of modulating immune-inflammatory and oxidative stress pathways with specificity and safety. The multidisciplinary approaches showcased in this Special Issue, particularly those integrating microbiota research, molecular profiling, and advanced imaging techniques, provide a solid foundation for future investigations aimed at improving disease management and patient outcomes.

Therefore, in this context, future research efforts should prioritize the validation of emerging biomarkers in well-designed clinical studies, the functional characterization and therapeutic targeting of immune-inflammatory and oxidative stress signaling pathways, and the refinement of patient stratification strategies to enable truly personalized interventions with the potential to notably improve patients’ quality of life.
